# Local Action with Global Impact: Highly Similar Infection Patterns of Human Viruses and Bacteriophages

**DOI:** 10.1128/mSystems.00030-15

**Published:** 2016-03-08

**Authors:** Rachelle Mariano, Sawsan Khuri, Peter Uetz, Stefan Wuchty

**Affiliations:** a Department of Computer Science, University of Miami, Coral Gables, Florida, USA; b Center for Computational Science, University of Miami, Coral Gables, Florida, USA; c Center for the Study of Biological Complexity, Virginia Commonwealth University, Richmond, Virginia, USA; MIT

**Keywords:** bacteriophages, host-pathogen interactions, protein interactions, viruses

## Abstract

The investigation of host-pathogen interaction interfaces and their constituent factors is crucial for our understanding of an organism’s pathogenesis. Here, we explored the interactomes of HIV, hepatitis C virus, influenza A virus, human papillomavirus, herpes simplex virus, and vaccinia virus in a human host by analyzing the combined sets of virus targets and human genes that are required for viral infection. We also considered targets and required genes of bacteriophages lambda and T7 infection in *Escherichia coli*. We found that targeted proteins and their immediate network neighbors significantly pool with proteins required for infection and essential for cell growth, forming large connected components in both the human and *E. coli* protein interaction networks. The impact of both viruses and phages on their protein targets appears to extend to their network neighbors, as these are enriched with topologically central proteins that have a significant disruptive topological effect and connect different protein complexes. Moreover, viral and phage targets and network neighbors are enriched with transcription factors, methylases, and acetylases in human viruses, while such interactions are much less prominent in bacteriophages.

**IMPORTANCE** While host-virus interaction interfaces have been previously investigated, relatively little is known about the indirect interactions of pathogen and host proteins required for viral infection and host cell function. Therefore, we investigated the topological relationships of human and bacterial viruses and how they interact with their hosts. We focused on those host proteins that are directly targeted by viruses, those that are required for infection, and those that are essential for both human and bacterial cells (here, *E. coli*). Generally, we observed that targeted, required, and essential proteins in both hosts interact in a highly intertwined fashion. While there exist highly similar topological patterns, we found that human viruses target transcription factors through methylases and acetylases, proteins that played no such role in bacteriophages.

## Abstract (precis)

While host-virus interaction interfaces have been previously investigated, relatively little is known about the indirect interactions of pathogen and host proteins required for viral infection and host cell function. Therefore, we investigated the topological relationships of human and bacterial viruses and how they interact with their hosts. We focused on those host proteins that are directly targeted by viruses, those that are required for infection, and those that are essential for both human and bacterial cells (here, *E. coli*). Generally, we observed that targeted, required, and essential proteins in both hosts interact in a highly intertwined fashion. While there exist highly similar topological patterns, we found that human viruses target transcription factors through methylases and acetylases, proteins that played no such role in bacteriophages.

## INTRODUCTION

The investigation of host-pathogen interactions and the factors required for pathogen infection represents a crucial step toward a thorough understanding of viral infections and provides a foundation for the development of effective means to prevent and combat infectious diseases. Recently, protein interaction interfaces of several human pathogens and their human host cells have been experimentally determined (1–7). Various RNA interference (RNAi) screens have additionally revealed sets of human proteins required by different human viruses to infect their host cells (8–10). Although these proteins do not necessarily physically interact with viral proteins, they play an indirect yet vital role in the infection process of many viruses.

The availability of sets of interacting human host and viral proteins has already prompted researchers to investigate the characteristics of these pathogen-host interfaces (11–19). Generally, human virus proteins tend to target hubs and bottleneck proteins in the underlying host protein interaction network. Cell cycle regulation, nuclear transport, and immune response proteins repeatedly emerged as prime targets that interact with different pathogens, suggesting that similar patterns to invade and manipulate important host processes exist.

Despite the abundance of analyses that cover various human viruses, such studies often focus entirely on the immediate host-pathogen interaction interface. In contrast, the relationships among the directly targeted host proteins, those required for effective infection regardless of physical interaction, and those essential for basal host cell function and survival remain poorly characterized. Given that large-scale patterns of different human-virus interaction interfaces feature significant similarities, we hypothesize that required gene sets may also manifest similar configurations across virus strains. To establish their relevance in different kingdoms, we further assume that analogous features may appear in bacteriophage-host interactions as well.

Here, we analyzed the topology of proteins targeted by HIV, hepatitis C virus, influenza A virus, herpes simplex virus, human papillomavirus (HPV), and vaccinia virus in addition to bacteriophages lambda and T7 in their corresponding host protein interaction networks. We found that targeted, required, and essential human and bacterial genes cluster in the immediate vicinity of directly targeted proteins, which suggests that these pathogens do not require extensive amplification through an interaction cascade to seize control of host cells. Additionally, targeted proteins form large connected subnetworks, while their immediate network neighbors are significantly enriched with proteins that are topologically central in the interaction network. Furthermore, targets and their immediate neighbors have a greater disruptive topological effect than randomly selected proteins and connect discrete protein complexes. Taken together, these results suggest that pathogen targets use their concentrated local impact to manipulate host function through subsequent access to a large and diverse fraction of host machinery (20). While transcription factors were enriched in the second-step network neighborhoods of targeted proteins in both hosts, methylases and acetylases significantly populated solely the first-step neighbors of human viruses. The observed need to access transcriptional activity through such proteins potentially reflects the higher epigenetic complexity of eukaryotes. Overall, our work indicates the existence of common infection patterns of pathogens in two dissimilar kingdoms.

## RESULTS

### Clustering of targeted and required proteins.

**FIG 1:**
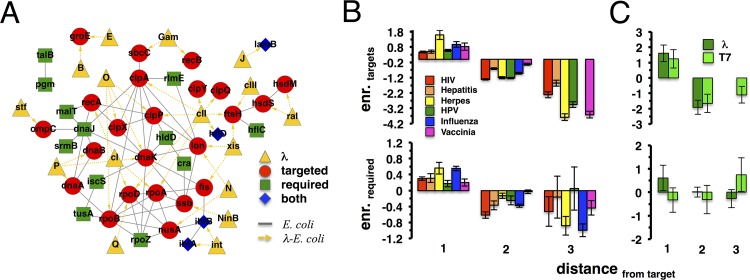
Targeted and required proteins cluster around targets of human and bacterial viruses. (A) Interactions between proteins of *E. coli* and bacteriophage lambda, accounting for proteins required for the infection process, as well as protein interactions between targeted and required genes, in *E. coli*. Notably, we found that such host interactions largely connected targeted and required genes. (B, C) At the top of panel B, we grouped human proteins that are a given shortest distance away from the nearest virus-targeted protein in the underlying protein-protein interaction network of *H. sapiens*. In each distance bin, we calculated the fraction of targeted proteins and randomly sampled targeted proteins as a null model. Notably, proteins targeted by human viruses strongly tend to interact with each other, a result that holds for bacteriophages lambda and T7 as well (top of panel C). At the bottom of each panel, we focused on genes that are required for the infection process of the underlying viruses. Remarkably, such proteins appeared predominantly in the network vicinity of targeted human and *E. coli* proteins.

We analyzed sets of *Escherichia coli* proteins targeted by proteins of bacteriophages lambda and T7 (21) and those required for the infection processes of each phage (22, 23) in the *E. coli* protein-protein interaction network (see Table S1 in the supplemental material). Figure 1A shows the subnetwork of interactions between lambda and *E. coli* proteins, suggesting that targeted and required proteins appeared to cluster in their own immediate network vicinity and created dense subnetworks in the underlying *E. coli* protein-protein interaction network. To quantify this trend, we determined the shortest path from each protein in the interaction network to the nearest protein that was targeted by a bacteriophage. In each distance bin, we calculated the enrichment of targeted proteins compared to a null model where we randomly selected targeted proteins from the *E. coli* interaction network and investigated their enrichment in the shortest paths between required and other targeted proteins. As shown at the bottom of Fig. 1B, we found that phage-targeted proteins indeed appeared to cluster strongly in their immediate network vicinity.

10.1128/mSystems.00030-15.7 Table S1  Overview of the human- and bacteriophage-specific targets and required proteins in this study. Download Table S1, PDF file, 0.02 MB. Copyright © 2016 Mariano et al. 2016 Mariano et al. This content is distributed under the terms of the Creative Commons Attribution 4.0 International license.

To test if the pattern observed applies to human viruses as well, we collected sets of human proteins targeted by HIV-1, herpes simplex virus, hepatitis C virus, influenza A virus, HPV-16, and vaccinia virus (24) (see Table S1 in the supplemental material). Importantly, these six human viruses are very different in taxonomy, nucleotide content, and mode of infection (see Table S2 in the supplemental material). In the top of Fig. 1B, we grouped human host proteins a given distance away from the nearest virus-specific targeted proteins, showing that targeted proteins frequently appeared in the immediate vicinity of other virus-targeted proteins. Furthermore, we collected sets of proteins that are required by HIV-1 (4, 8, 25), herpes simplex virus (26), hepatitis C virus (10), HPV-16 (27), influenza A virus (3, 9, 28, 29), and vaccinia virus (30) (see Table S1 in the supplemental material) to successfully invade a host cell. Figure 1C shows similar clustering trends of required proteins around targeted proteins (top), observations that also hold for required proteins of bacteriophages (bottom).

10.1128/mSystems.00030-15.8 Table S2  Overview of the human viruses in this study. Download Table S2, PDF file, 0.1 MB. Copyright © 2016 Mariano et al. 2016 Mariano et al. This content is distributed under the terms of the Creative Commons Attribution 4.0 International license.

**FIG 2:**
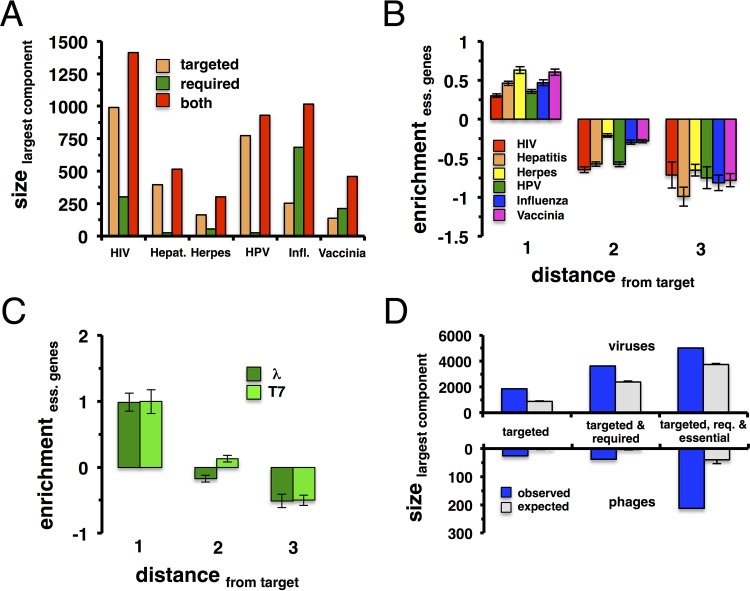
Proteins that are targeted and required by human viruses/bacteriophages and essential proteins form large connected components. (A) We determined the largest connected component of interactions between proteins that were targeted and/or required by a given human virus. As a null model, we randomly sampled such sets, showing that the size of such large connected components is statistically significant (*P* < 10^−4^). (B) Determining the enrichment of essential human genes that are topologically placed a given distance away from the nearest viral targets, we found that essential genes cluster around viral targets. (C) We obtained a similar result when we considered essential *E. coli* genes. (D) We also pooled all human virus targets and augmented such a set with required and essential human genes that interact with viral targets. Interactions between such proteins formed large connected components (*P* < 10^−4^). We obtained a similar result when we pooled proteins that are targeted and required by bacteriophages, as well as essential genes in *E. coli* (*P* < 10^−4^).

The clustering tendency of targeted and required proteins suggests that these proteins may form large connected components in the underlying protein interaction networks. Considering all human interactions between HIV-targeted proteins, we found that 991 out of 997 targeted proteins formed a connected component through their mutual protein interactions (Fig. 2A). Random samples of 997 proteins from the human interactome yielded much smaller sizes of the corresponding connected components, showing that HIV-targeted proteins are significantly tied together (*P* < 10^−4^). These observations held true for the remaining viruses, where roughly >95% of the targeted proteins assembled the largest connected components (Fig. 2A). By the same token, we determined the sizes of the largest connected components that were composed of interactions between required proteins. While we observed similarly significant behavior (*P* < 10^−4^), such subnetworks generally assembled a lesser fraction of interacting required proteins than their targeted counterparts. In particular, >95% of the interacting genes that were required by HIV and influenza virus formed the largest connected components, while we found roughly 80% for vaccinia virus, 50% for hepatitis C virus and herpesvirus, and 33% for HPV. When we considered connected components of both targeted and required genes, >90% of these combined protein sets formed the largest connected components. We found a comparable result when we considered the largest components of genes that were targeted and/or required by bacteriophage lambda (see Fig. S1 in the supplemental material), where the largest subnetwork was composed of 17 (85%) out of 20 targeted proteins. While only 3 proteins formed the largest component of required proteins, we found a large component of 27 (>80%) out of 33 proteins when we considered targeted and required proteins of bacteriophage lambda.

10.1128/mSystems.00030-15.1 Figure S1  Genes that are targeted and required by bacteriophage lambda are organized in a large connected component. We determined the largest connected component of interactions between *E. coli* proteins that were targeted and/or required by bacteriophage lambda. As a null model, we randomly sampled such sets, suggesting that targeted and/or required proteins appeared to be organized in large subnetworks (*P* < 10^−4^). Download Figure S1, PDF file, 0.1 MB. Copyright © 2016 Mariano et al. 2016 Mariano et al. This content is distributed under the terms of the Creative Commons Attribution 4.0 International license.

### Essential genes.

Previous work indicates that essential human genes are enriched in many different diseases (31), prompting us to investigate their topological role in the presence of pathogens. Utilizing a set of 712 essential *E. coli* genes (32) we observed that essential genes were significantly targeted by bacteriophages (*P* < 0.02 [Fisher exact test]). We obtained a similar result by using 2,708 human essential genes (*P* < 5 × 10^−5^ [Fisher exact test]) (33). Such a result may reflect the need of pathogens to completely redirect basic processes to their own propagation. Hypothesizing that essential genes cluster in the vicinity of pathogen-targeted proteins, we indeed found that they were enriched in bins of proteins located a given distance away from the nearest target in the underlying human and *E. coli* protein interaction networks (Fig. 2B and C). Given their clustering characteristics, essential proteins in the vicinity of viral targets may also contribute to the formation of a large connected component. In the top of Fig. 2D, we show that the pool of all genes targeted by at least one human virus forms a significantly large connected component when we randomly sampled sets of targets (*P* < 10^−4^). Since required genes preferably cluster in the immediate vicinity, we augmented this set with proteins that were required by at least one virus and interacted with at least one viral target, allowing us to find a significantly large connected component as well (*P* < 10^−4^). Accounting for interactions between human and bacterial essential proteins, we observed large connected components as well (see Fig. S2 in the supplemental material), a result that corroborates observations on other organisms (34). Combining sets of targeted, required, and essential proteins connected to pathogen targets, we attained an even bigger, significantly large connected component (*P* < 10^−4^). As for bacteriophages, we observed a similar, albeit weaker, signal when we considered pools of phage targets, as well as interacting required and essential proteins (Fig. 2D, bottom). Such a result may be the consequence of the comparatively much larger set of essential proteins of *E. coli* that not only form a substantially large connected component but also overshadow the connected component of interactions between phage-targeted and required proteins (34).

10.1128/mSystems.00030-15.2 Figure S2  Essential genes in *H. sapiens* and *E. coli* are organized in a large connected component. We determined the largest connected component of interactions among essential human and bacterial genes. As a null model, we randomly sampled such sets, suggesting that essential genes appeared to be organized in large subnetworks (*P* < 10^−4^). Download Figure S2, PDF file, 0.1 MB. Copyright © 2016 Mariano et al. 2016 Mariano et al. This content is distributed under the terms of the Creative Commons Attribution 4.0 International license.

### Biological functions.

**FIG 3:**
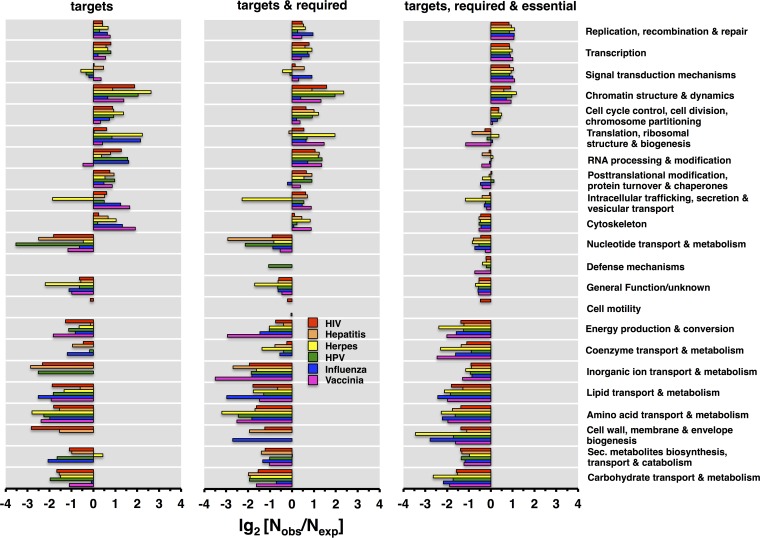
Functional classes of human virus targets and their neighboring required and essential genes in *H. sapiens*. Pooling virus-specific targeted, as well as required and essential, human proteins that interact with viral targets, we determined the numbers of proteins that belong to the underlying functional COG classes. As a random null model, we resampled proteins in such classes 10,000 times.

Reflecting the close relationship between targeted, required, and essential proteins that interact with pathogen targets, we investigated their functional consequences by using a cluster of orthologous groups (COG)-based classification of proteins (35). Human-specific virus-targeted, required, and essential genes show similar enrichment patterns in their immediate vicinity (Fig. 3). Notably, the successive addition of required and essential genes to targeted proteins provided more homogeneous enrichment patterns, suggesting that such genes were found predominantly in the chromatin structure and dynamics, cell cycle control, transcription, replication, and signal transduction categories. We obtained a similar result when we considered enrichment/depletion patterns of corresponding sets of bacteriophage-targeted, required, and essential *E. coli* genes (see Fig. S3 in the supplemental material).

10.1128/mSystems.00030-15.3 Figure S3  Functional classes of bacteriophage-targeted, as well as neighboring required and essential, genes in *E. coli*. Pooling bacteriophage-specific targeted proteins and their interacting required and essential *E. coli* proteins, we determined the numbers of proteins that belong to the underlying functional COG classes. As a random null model, we resampled proteins in such classes 10,000 times. Download Figure S3, PDF file, 0.1 MB. Copyright © 2016 Mariano et al. 2016 Mariano et al. This content is distributed under the terms of the Creative Commons Attribution 4.0 International license.

### Centrality.

**FIG 4:**
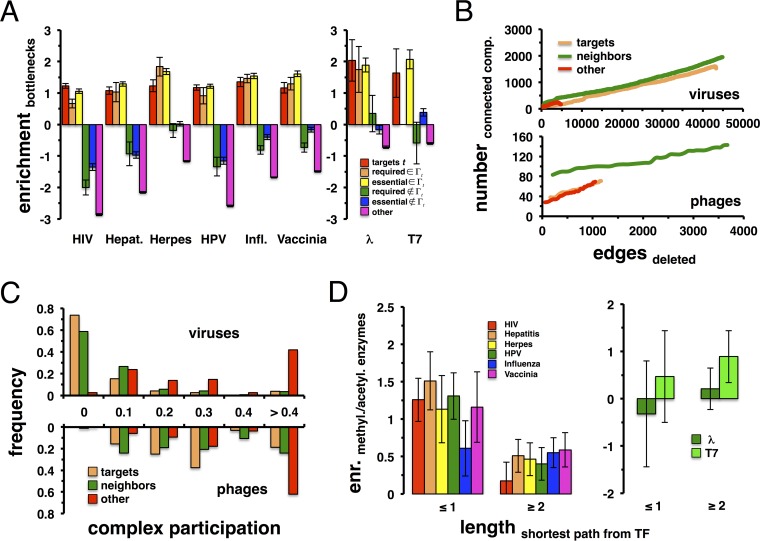
Centrality of targeted and neighboring proteins. (A) We considered virus-specific targets (*t*), as well as required and essential genes, that (do not) appear in the network vicinity of targeted proteins Γ_t_. We defined a set of human bottleneck proteins as the top 20% of proteins with the highest betweenness centrality in the underlying human protein interaction network. In such a set, virus-specific targets, as well as required and essential genes in the vicinity of viral targets, appeared enriched. Notably, other required, essential, and remaining proteins appeared significantly noncentral. Analogously, we obtained similar results by considering proteins that were targeted and required by phages, as well as essential *E. coli* genes. (B) Considering human targets of all viruses, we ordered such proteins according to their degree in the underlying human interaction network. Successively removing proteins, we calculated the number of connected components after each deletion step. In comparison, we investigated the disruptive impact of protein sets of equal size that were composed of target neighbors and remaining proteins. Notably, we observed that the deletion of proteins that are placed in the network neighborhood of targets had the most destructive effect. Such a result also holds for targets and neighboring proteins of bacteriophages. (C) As a different measure of centrality, we calculated the complex participation coefficient, reflecting a protein’s ability to span different protein complexes through its interactions. Considering targeted proteins and their immediate network neighbors, we observed that corresponding frequency distributions peak at low participation values. In turn, remaining proteins appear at higher values, suggesting that targets and their neighbors predominantly reach different complexes through their interactions in both hosts. (D) We identified the shortest paths from human transcription factors to their nearest viral targets and determined the enrichment of human acetylation/methylation enzymes in paths of given lengths. Randomly sampling such protein sets, we observed that viruses predominantly targeted such enzymes and kinases as to indirectly interact with transcription factors. As shown in the inset, we observed the opposite when we considered the enrichment of such enzymes in paths from transcription factors in *E. coli* to the nearest phage-targeted protein.

Previous studies indicated that pathogens tend to target centrally located host proteins. To verify this assumption, we quantified the betweenness centrality of each protein, defined as the sum of the fraction of occurrences where the protein in question inhabits the shortest path between any two other proteins in the network. Calculating the betweenness centrality of proteins in *Homo sapiens*, we defined the top 20% of the most central proteins as “bottleneck proteins.” Pooling virus-targeted proteins, we determined their enrichment in these sets of highly central proteins and confirmed that viruses tend to target these bottleneck proteins (Fig. 4A). In comparison, we repeated our analysis with required and essential genes that interacted with targeted proteins, allowing us to find that these sets of proteins were enriched with bottleneck proteins as well. When we considered the sets of required and essential genes that did not directly interact with targets, we surprisingly found a strong dilution of bottleneck proteins, a result that was confirmed when we considered bottleneck proteins in *E. coli*.

To measure a protein’s impact on an interaction network’s resilience, we performed a robustness analysis. We sorted all of the targeted proteins of human viruses according to their degrees in the underlying human interaction network. Starting with the most connected protein, we gradually deleted proteins and calculated the number of disconnected subnetworks in the remaining protein interaction network after each deletion step. In comparison to the set of viral targets, we considered sets of equal size of proteins that interact with targeted proteins and remaining proteins, respectively. Repeating our analysis, the top of Fig. 4B indicates that the successive deletion of neighboring proteins had a greater disruptive impact on network topology by creating more connected components than direct targets. Notably, we observed a strong reinforcement of this type of trend when we considered neighbors of phage targets in *E. coli* phages (Fig. 4B, bottom).

### Protein complexes.

As a different measure of centrality, we determined a protein’s propensity to interact with numerous protein complexes through their interactions. Such a complex participation coefficient shifts to 0 when a given protein reaches proteins in many different complexes and to 1 if it interacts with proteins of the same complex (13). Using 1,843 human protein complexes (36), we considered targeted proteins of all viruses and calculated their corresponding complex participation coefficients (Fig. 4C, top; see Fig. S4 in the supplemental material). Furthermore, we also accounted for corresponding distributions of proteins that were placed in the immediate vicinity of viral targets, indicating that these proteins appear to reach many different protein complexes as well. In turn, remaining proteins that did not interact with the set of virus targets interacted mostly with proteins in the same protein complexes. For bacteriophages, we utilized a set of 517 protein complexes in *E. coli* (37), allowing us to obtain similar, albeit weaker, results (Fig. 4C, bottom; see Fig. S4 in the supplemental material).

10.1128/mSystems.00030-15.4 Figure S4  Complex participation of targeted, neighboring, and other human or *E. coli* proteins. We considered proteins that were targeted by a given virus or bacteriophage, as well as their immediate network neighbors. Calculating their complex participation coefficient, we observed that such sets of proteins predominantly reached into many different complexes, while sets of remaining proteins preferably interacted with proteins in the same complexes. Download Figure S4, PDF file, 0.2 MB. Copyright © 2016 Mariano et al. 2016 Mariano et al. This content is distributed under the terms of the Creative Commons Attribution 4.0 International license.

### Transcriptional proteins.

Since targeted and required proteins form large connected components in the host protein interaction networks, we assumed that the viruses directly impact their hosts through interactions in the immediate vicinity of the primary targets. In particular, pathogens may utilize the immediate vicinity of their protein targets to gain control of transcription factors as the primary lever to control host protein expression. As shown in Fig. S5 in the supplemental material, we indeed found that transcription factors appeared enriched in sets of viral protein targets and network neighbors. Furthermore, we observed that such a set was enriched for acetylation and methylation proteins, a result that roughly holds for phage targets and their neighbors as well (see Fig. S5 in the supplemental material). The presence of such proteins in the set of targets and neighbors suggests that methylation and acetylation enzymes may allow the pathogens to reach or influence transcription factor activity. In particular, we observed that the shortest paths from human and bacterial transcription factors to their nearest virus- or phage-targeted genes were significantly shorter than randomly sampled sets of targeted genes (*P* < 0.05, Student’s *t* test; see Fig. S6 in the supplemental material). Determining the enrichment of human acetylation and methylation enzymes in the observed shortest paths from transcription factors, we found that viruses preferably targeted these proteins to interact with transcription factors (Fig. 4D). In turn, direct targets of bacteriophages were rarely methylases or acetylases (Fig. 4D), suggesting that methylation and acetylation play little or no important role in transcriptional interference by coliphages.

10.1128/mSystems.00030-15.5 Figure S5  Enrichment of transcription factors and methylation/acetylation enzymes. Considering sets of virus/phage targets and their immediate network neighbors, we observed that transcription factors and acetylation/methylation enzymes appear enriched in such sets. In turn, such proteins were depleted in remaining sets of proteins. Download Figure S5, PDF file, 0.1 MB. Copyright © 2016 Mariano et al. 2016 Mariano et al. This content is distributed under the terms of the Creative Commons Attribution 4.0 International license.

10.1128/mSystems.00030-15.6 Figure S6  Frequency distributions of shortest paths from transcription factors to nearest targeted proteins. (A) We calculated the shortest path in a human interaction network from each human transcription factor to the nearest protein that was targeted by a given virus. Randomly sampling such sets of targeted proteins, we observed that such paths were significantly shorter than their random counterparts (*P* < 0.03 [Student’s *t* test]). (B) We obtained similar results when we considered the shortest path lengths from transcription factors in *E. coli* to the nearest phage-targeted proteins (*P* < 10^−10^ [Wilcoxon test]). Download Figure S6, PDF file, 0.1 MB. Copyright © 2016 Mariano et al. 2016 Mariano et al. This content is distributed under the terms of the Creative Commons Attribution 4.0 International license.

## DISCUSSION

Our analysis demonstrates that required and essential host proteins are generally found an immediate interactive distance from targeted proteins. As a consequence, such sets of targeted proteins, as well as required and essential proteins that interact with pathogen targets, form large connected components in the underlying human and *E. coli* protein interaction networks. These findings indicate a potentially large host-pathogen interface that extends beyond directly targeted proteins and allows pathogens to obtain direct access to the underlying host through several pathways. Notably, the sizes of connected components that were composed of targets and required gene sets of different viruses varied greatly. While such differences may indicate underlying differences of corresponding virus-host interactomes, they also may be a consequence of their incomplete experimental determination. Despite such shortcomings, host-pathogen interactomes reflected a high degree of functional similarity, indicating the enrichment of certain functions. Notably, the addition of required and essential genes provided more homogeneous functional enrichment patterns. By influencing key regulatory functions such as RNA processing, chromatin remodeling, cell cycle control, transcription, replication, and signal transduction, proteins that are in close proximity to targeted genes may act critically during pathogen infection by rapidly assuming control of host gene expression. Recent research supports this view, indicating that genes topologically close to disease genes can be potentially disease relevant (38). Furthermore, proteins targeted by pathogens, as well as their immediate network neighbors, allow the pathogen to reach numerous protein complexes, not only indicating a pool of responsive candidate genes for a single virus/phage to influence but also suggesting a host-pathogen interface model that permits the pathogens to quickly take control of the underlying host cell. Moreover, we found that neighbors of targets have an even greater disruptive effect on the underlying topology of host protein interaction networks than directly targeted proteins. Such a result was especially pronounced when we considered neighbors of *E. coli* proteins that were targeted by bacteriophages. While we found a similar yet less significant result in the human interactome, we assume that such observations are rooted in the relatively small phage-host interaction interface. In particular, most viral targets were determined by using high-throughput techniques while phage-host interactions were collected primarily from low-throughput studies. Since high-throughput screens tend to include a certain fraction of false positives, such interactions may attenuate detectable effects.

Furthermore, we found that transcription factors, as well as methylation and acetylation enzymes, are enriched among targets and immediate neighbors and appear strongly diluted in sets of remaining genes in both hosts. These enzymes appear to be direct gateways for human viruses to interact with transcription factors, suggesting that epigenetic changes may expedite human viral infections. In contrast, we found the opposite when we considered bacteriophages, indicating that epigenetic processes may not overtly mediate bacteriophage infection. While we note that fewer methylation and acetylation enzymes exist in *E. coli*, methylases are involved in RNA rather than protein methylation. While RNA modification is considered to play a role in phage infection, these pathways are poorly understood (39), suggesting that epigenetic effects may play a relatively minor role for bacteriophages (40).

Given the rapid evolution of bacteriophages, different species appear to use very different strategies. For instance, phage lambda interacts with several host proteases, which seems to be rare in T7 (41). However, *E. coli* and human interactomes are as yet incomplete, with the human system being less well understood and an order of magnitude more complicated than microbial systems. While the infection patterns of bacteriophages and viruses appear similar, we cannot rule out the possibility that the observed difference is a consequence of incomplete data rather than a true difference between viruses and phages.

In conclusion, therefore, once a virus gains access to a host cell, it quickly gains local control over the host system by engaging a large network of closely interconnected genes that are targeted directly, required indirectly, or part of the essential protein machinery of the host. This observation has a global impact, being true in a varied selection of human viruses, as well as bacteriophages. We expect protein interaction data from other host-viral systems to verify this trend as more such data become available, indicating universal mechanisms of viral infection and pathogenesis.

## MATERIALS AND METHODS

### Protein-protein interaction data.

We collected a total of 11,463 interactions between 2,765 proteins in *E. coli* (37, 42, 43). As for *H. sapiens*, we used 70,124 interactions between 12,801 human proteins that were collected from the HINT (44), MINT (45), BioGrid (46), and HPRD (47) databases. For both organisms, we considered binary, as well as cocomplex, interactions.

### Essential genes.

We used 712 essential proteins in *E. coli* from the database of essential genes DEG10, an update of the database of essential genes (DEG) that collects data about essential genes from the literature (32). For human proteins, we utilized 2,708 essential genes that were determined by massively parallel RNAi screening (33).

### Bacteriophage-host interactions.

We collected 27 *E. coli* proteins that were involved in interactions with lambda proteins detected by a yeast two-hybrid approach (21). In turn, we utilized 16 *E. coli* proteins collected from the literature (21) that were interacting with T7 proteins (41). We used a set of 57 genes of *E. coli* that were required for lambda infection (22). Furthermore, we utilized 11 genes of *E. coli* that were required for T7 infection of the host (23) (Fig. 1B). In both cases, the effect of these genes on the replication of the corresponding phages was experimentally assessed when they were knocked out in *E. coli.*

### Virus-host interactions.

Collecting data from the HPIDB database, we used 697 human proteins that were targeted by hepatitis C virus, as well as 255 targets of herpes simplex virus, 1,272 targets of HIV-1, 396 targets of influenza A virus, and 317 targets of vaccinia virus (24) (Fig. 1B). We used 262 genes that were required by hepatitis C virus to infect a human host cell (10). For herpes simplex virus, we collected 358 such genes (26). Furthermore, we utilized 917 such genes of HIV-1 (4, 8, 25), 1,101 genes of vaccinia virus (30), and 1,251 genes of influenza A virus (3, 9, 28, 29) (Fig. 1B).

### Transcription factors and acetylation and methylation proteins.

We collected 1,572 human transcription factors from the DBD database (48) and 257 *E. coli* transcription factors from the EcoCyc database (49). Moreover, we collected 570 human and 167 methylation and acetylation enzymes in *E. coli* from the UniProt database (50).

### Enrichment analysis.

Binning proteins with a certain characteristic *d* (e.g., being a certain distance away from a reference protein), we calculated the fraction of proteins that had a feature *i* in each group *d*, *f_i_*(*d*). As a null model, we randomly sampled protein sets with feature *i* of the same size 10,000 times and calculated the corresponding random fraction, *f*_i,*r*_(*d*). The enrichment/depletion of proteins with feature *i* in a group *d* was then defined as *E_i_*(*d*) = log_2_[*f_i_*(*d*)/*f*_i,*r*_(*d*)].

### Protein complexes in *E. coli* and *H. sapiens*.

For *E. coli*, we utilized a set of 517 protein complexes from a coaffinity purification/mass spectrometry study (37). For human data, we utilized 1,843 protein complexes from the Corum database (36).

### Bottleneck proteins.

As a global measure of its centrality, we defined the betweenness centrality *c_B_* of a protein *v* as

$$c_{B}\left(v\right)=\sum_{s\neq t\neq v\in V}\frac{\sigma_{st}\left(v\right)}{\sigma_{st}}$$

where *σ*_*st*_ is the number of shortest paths between proteins *s* and *t* while *σ*_*st*_(*v*) is the number of shortest paths running through protein *v*. As a representative set of bottleneck proteins, we selected the top 20% of the most central proteins.

### Functional classes of proteins.

*E. coli* and *H. sapiens* proteins were grouped according to broad functional classes that were defined by COGs (35). Specifically, COGs provide a consistent classification of bacterial and eukaryotic species based on orthologous groups.

### Protein complex participation coefficient.

For each protein that is part of at least one protein complex, we defined the protein complex participation coefficient of a protein *i* as

$$P_{i}=\sum_{s=1}^{N}\left(n_{i,s}/\sum_{s=1}^{N}n_{i,s}\right)$$

where *n*_i,*s*_ is the number of links protein *i* has to proteins in complex *s* out of a total of *N* complexes. If a protein interacts predominantly with partners of the same complex, *P* tends to 1. In turn, *P* tends to 0 if a protein interacts with partners in a variety of protein complexes (13).
