# Supplementary material for: Local Action with Global Impact: Highly Similar Infection Patterns of Human Viruses and Bacteriophages
Source: mSystems. 2016 Mar 8;1(2):e00030-15. doi: 10.1128/mSystems.00030-15 (PMC5069743; doi:10.1128/mSystems.00030-15)
Supplement: Table S1 [file sys002162007st1.pdf]

	viruses	
	targeted	required
HIV-1	1,272	917
Herpes	255	358
Hepatitis C	697	262
HPV	1,020	315
Influenza A	396	1,251
Vaccinia	317	1,101

	bacteriophages	
	targeted	required
λ	27	57
T7	16	11
