# Supplementary material for: Local Action with Global Impact: Highly Similar Infection Patterns of Human Viruses and Bacteriophages
Source: mSystems. 2016 Mar 8;1(2):e00030-15. doi: 10.1128/mSystems.00030-15 (PMC5069743; doi:10.1128/mSystems.00030-15)
Supplement: Table S2 [file sys002162007st2.pdf]

	Taxonomy	Genome	Transmission
**HIV-1**	Retrovirus/lentivirus	2x neg ss RNA	Body fluid
**Herpes simplex**	Herpesviridae	ds linear DNA	Direct transfer
**Hepatitis C**	Flaviviridae	pos ss RNA	Blood-borne
**HPV 16**	Papillomaviridae	ds linear DNA	Sexual contact
**Influenza A**	Orthomyxoviridae	neg ss RNA	Saliva droplets
**Vaccinia**	Poxvirus	ds linear DNA	Direct transfer
