# Supplementary figures and images for: Local Action with Global Impact: Highly Similar Infection Patterns of Human Viruses and Bacteriophages
Source: mSystems. 2016 Mar 8;1(2):e00030-15. doi: 10.1128/mSystems.00030-15 (PMC5069743; doi:10.1128/mSystems.00030-15)

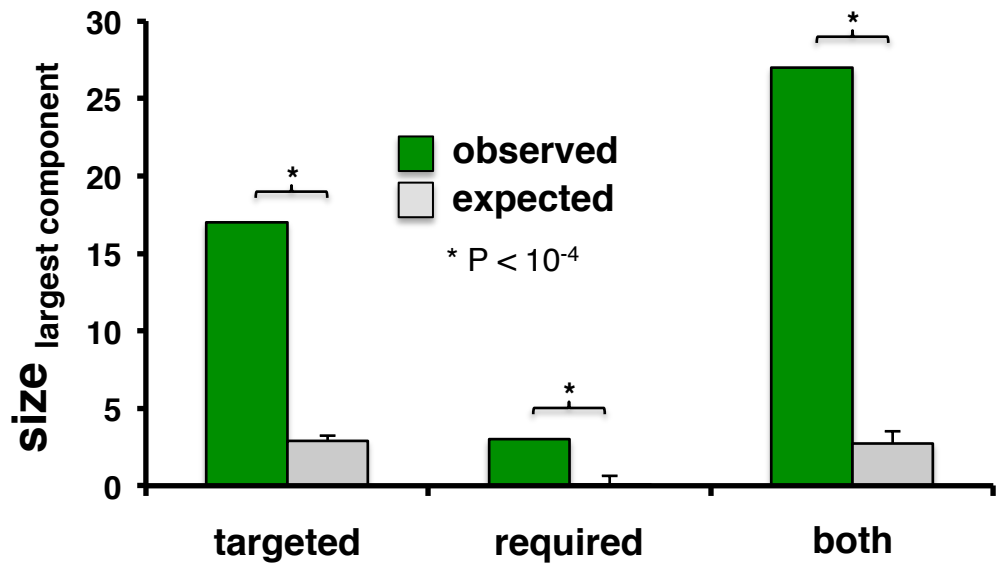

Supplement: Figure S1 [file sys002162007sf3.pdf]

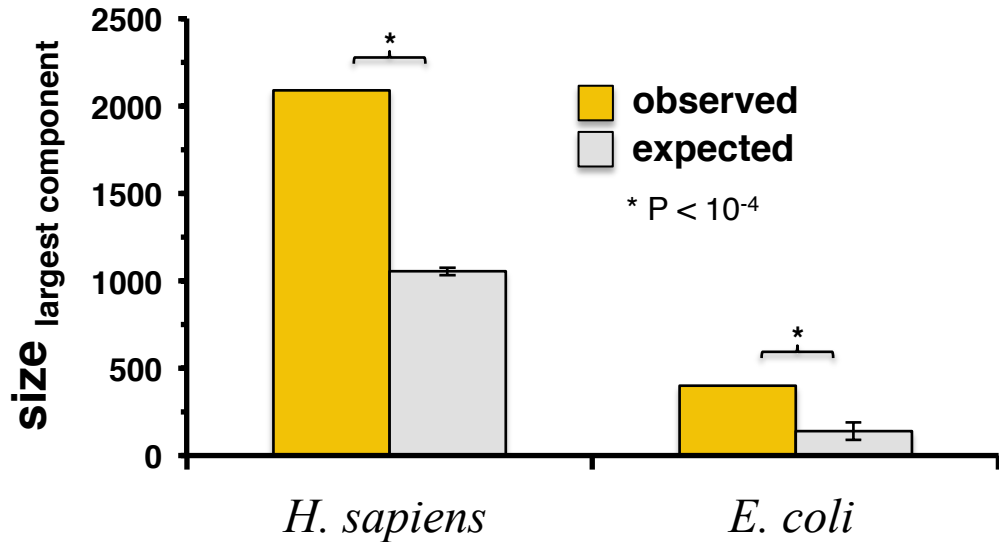

Supplement: Figure S2 [file sys002162007sf4.pdf]

targets

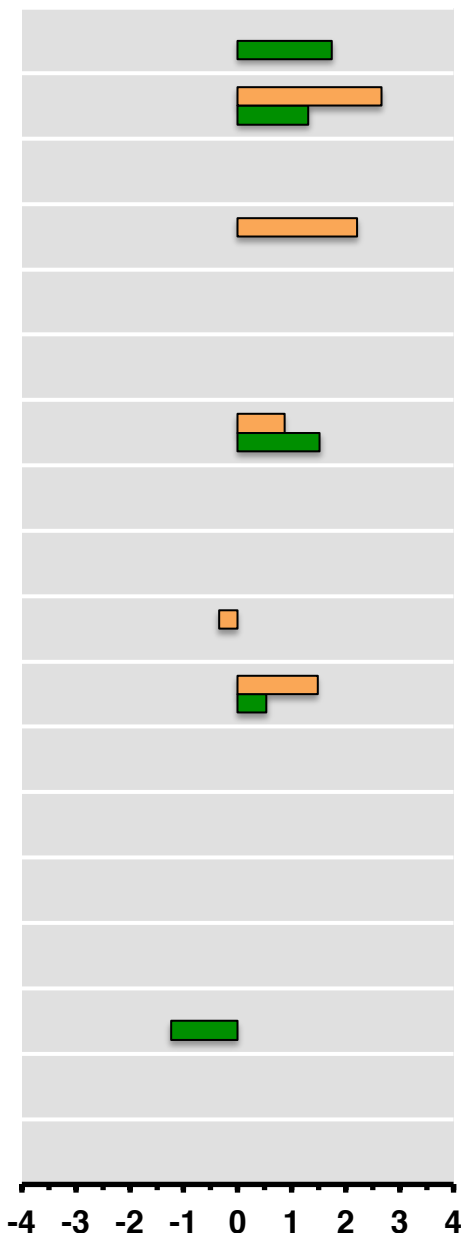

targets & required

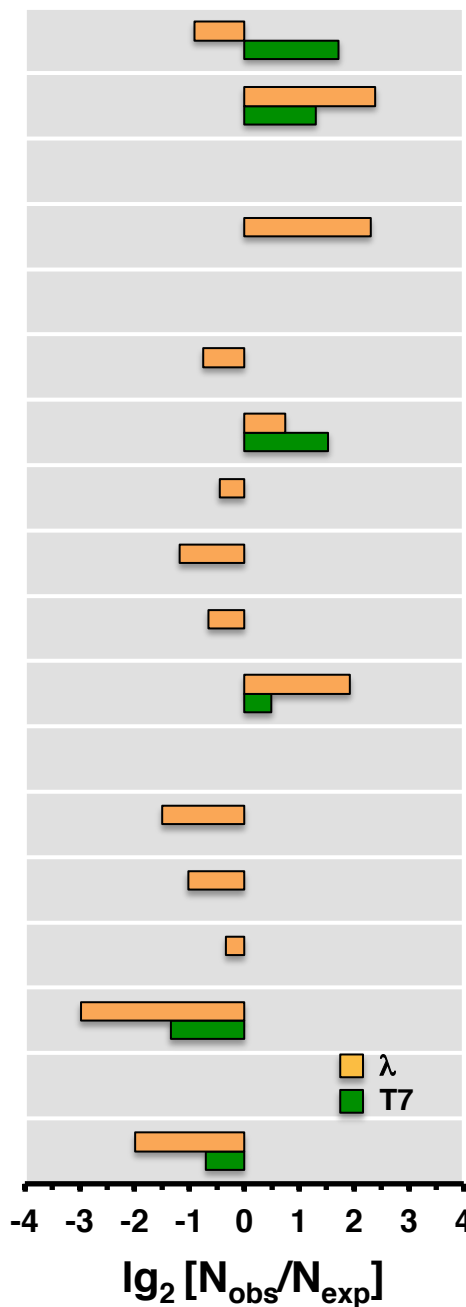

targets, required & essential

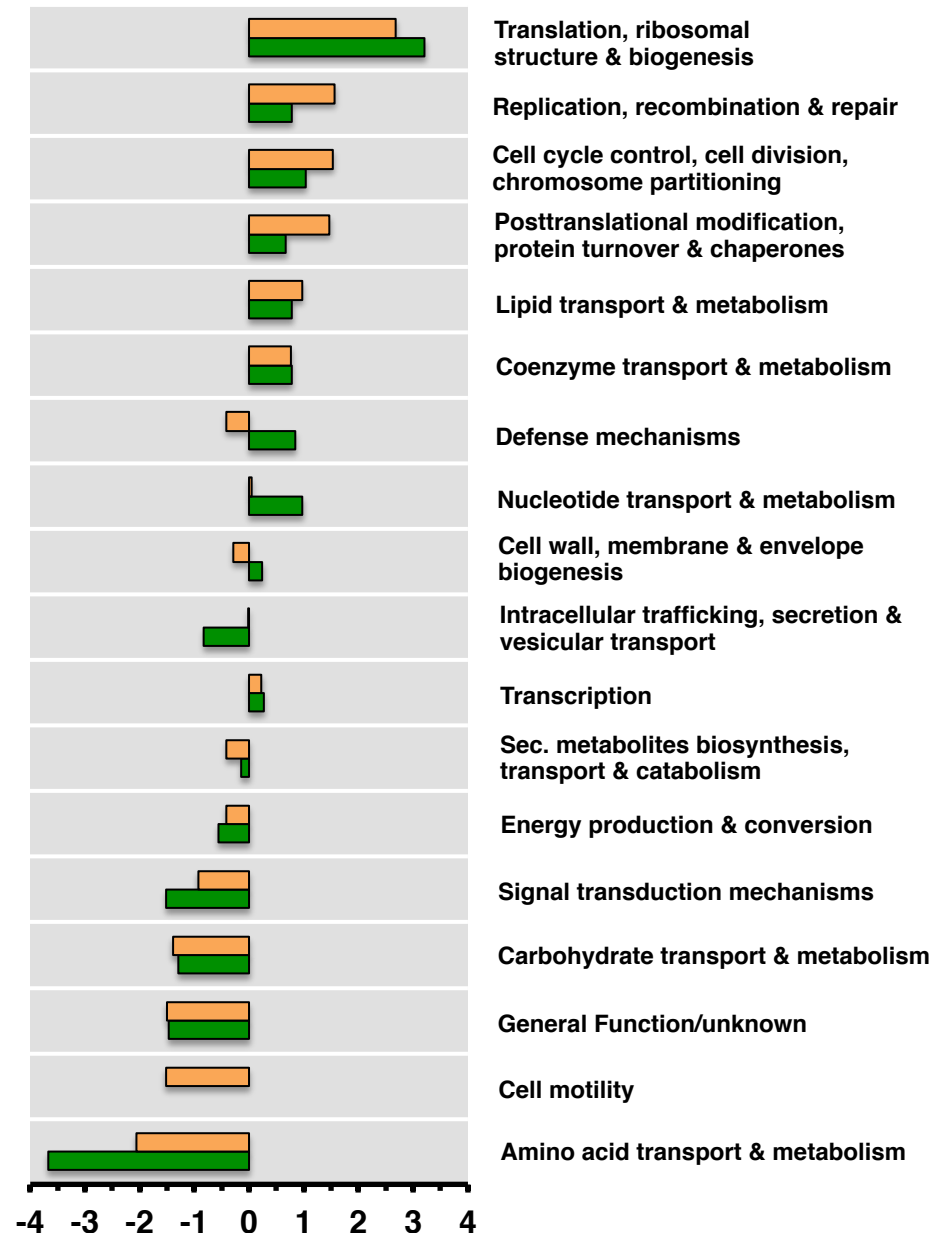

Supplement: Figure S3 [file sys002162007sf5.pdf]

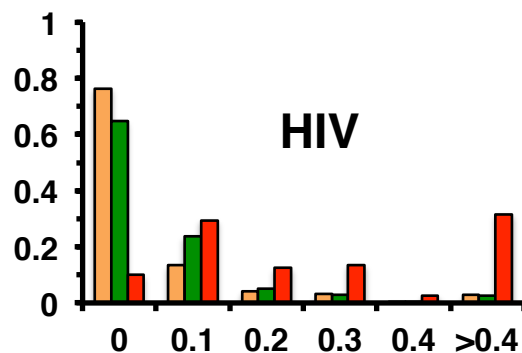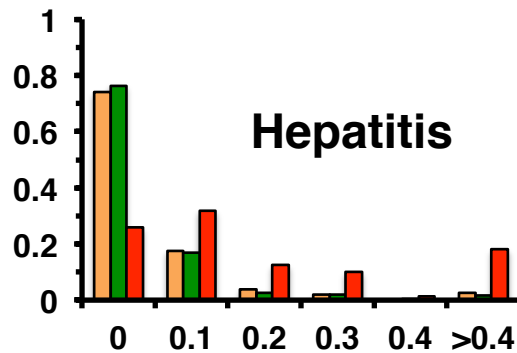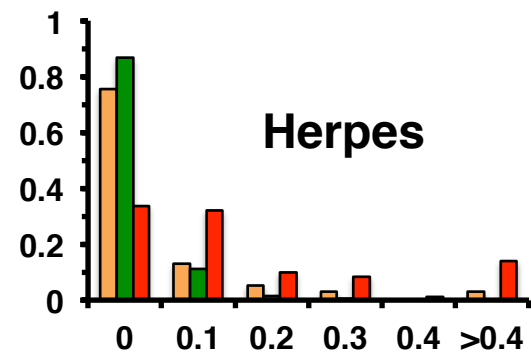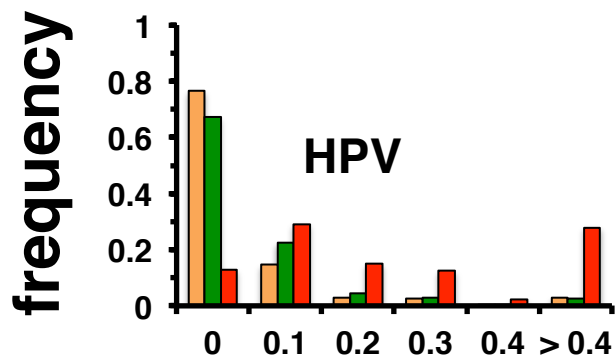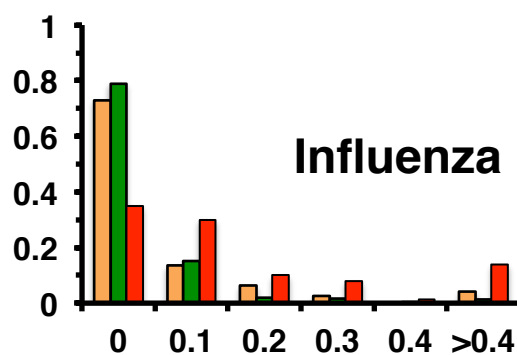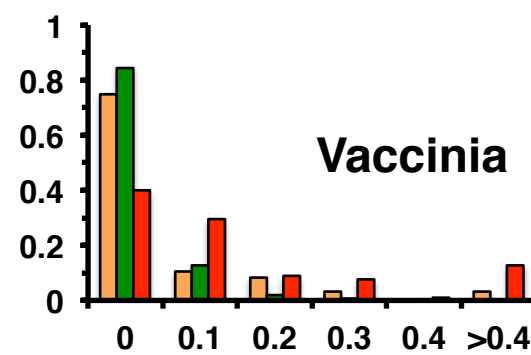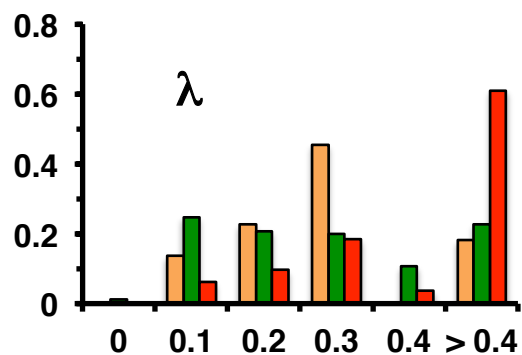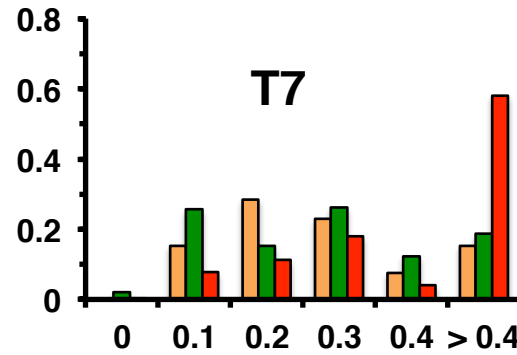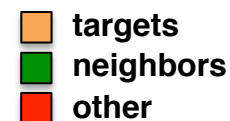

participation complex

Supplement: Figure S4 [file sys002162007sf6.pdf]

enrichment  
legends

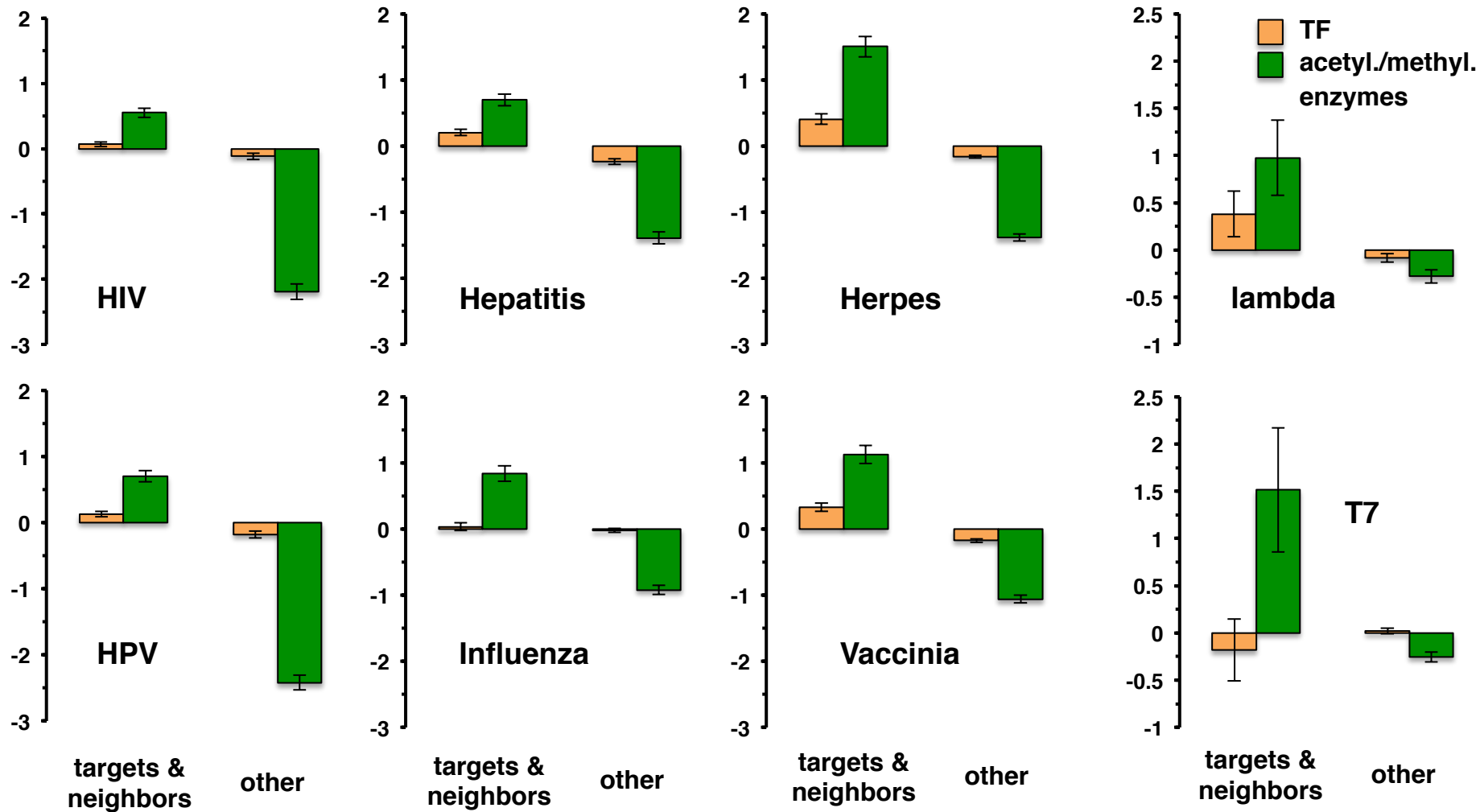

Supplement: Figure S5 [file sys002162007sf7.pdf]

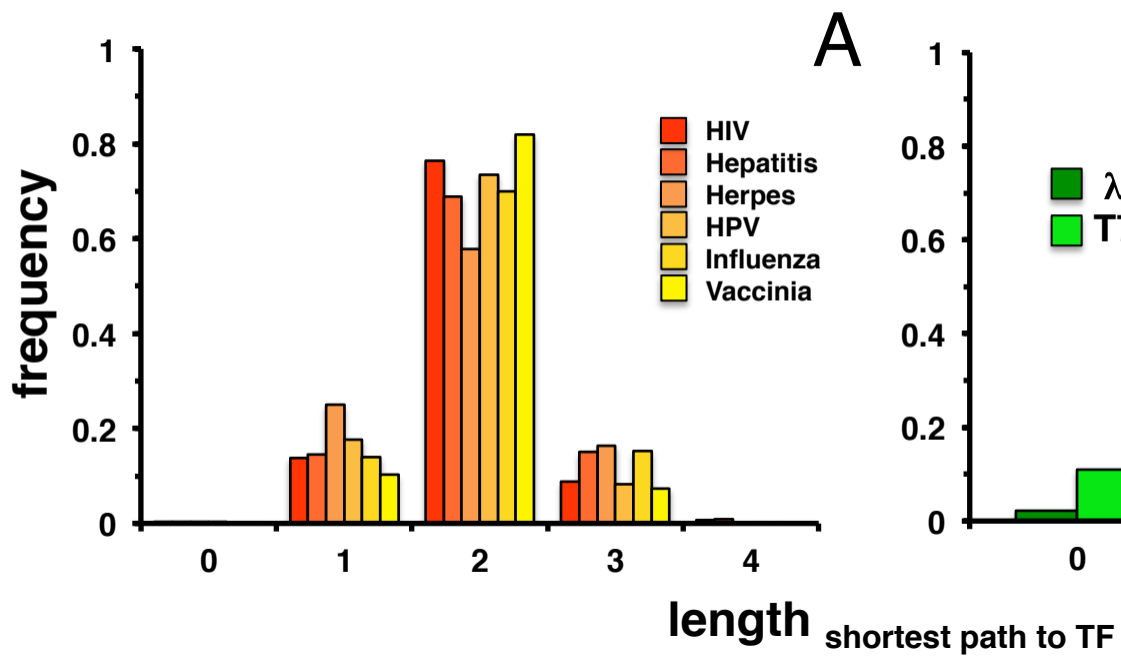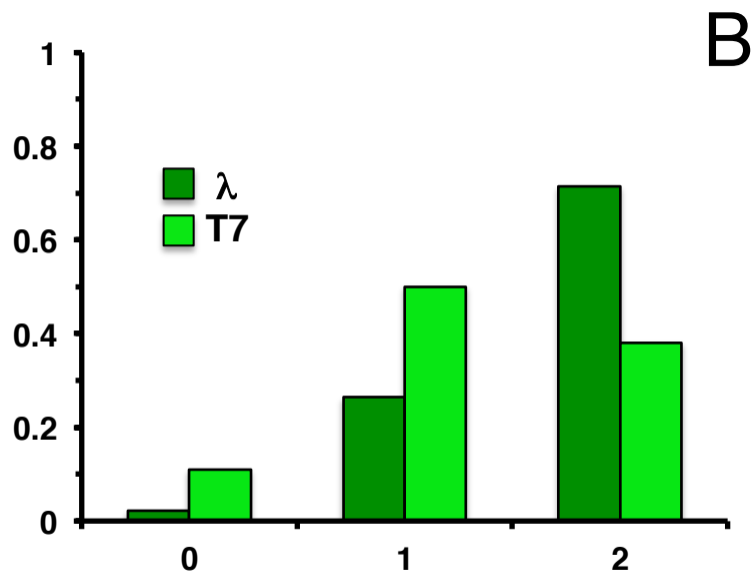

Supplement: Figure S6 [file sys002162007sf8.pdf]
